# Investigation of the Environmental Stability of Poly(vinyl alcohol)–KOH Polymer Electrolytes for Flexible Zinc–Air Batteries

**DOI:** 10.3389/fchem.2019.00678

**Published:** 2019-10-22

**Authors:** Xiayue Fan, Jie Liu, Jia Ding, Yida Deng, Xiaopeng Han, Wenbin Hu, Cheng Zhong

**Affiliations:** ^1^Key Laboratory of Advanced Ceramics and Machining Technology (Ministry of Education), School of Materials Science and Engineering, Tianjin University, Tianjin, China; ^2^Tianjin Key Laboratory of Composite and Functional Materials, School of Materials Science and Engineering, Tianjin University, Tianjin, China; ^3^Joint School of National University of Singapore and Tianjin University, International Campus of Tianjin University, Fuzhou, China

**Keywords:** poly(vinyl alcohol), KOH, gel polymer electrolyte, environmental stability, flexible zinc–air batteries

## Abstract

Next-generation wearable and portable electronic devices require the development of flexible energy-storage devices with high energy density and low cost. Over the past few decades, flexible zinc–air batteries (FZABs), characterized by their extremely high theoretical energy density from consuming oxygen in air and low cost, have been regarded as one of the most promising power supplies. However, their unique half-open structure poses great challenges for the environmental stability of their components, including the electrolyte and electrodes. As an important ionic conductor, the poly(vinyl alcohol) (PVA)–KOH gel polymer electrolyte (GPE) has been widely utilized in FZABs. To date, most studies have focused on investigations of the electrode, electrocatalyst materials and battery configuration, while very few have paid attention to the influence of the environment on the electrolyte and the corresponding FZAB performance. Herein, for the first time, the environmental stability of PVA–KOH GPE, such as dimensional stability and water and ionic conductivity retention capability, for FZABs in ambient air has been thoroughly studied. Moreover, the properties of the assembled FZABs in terms of cycling stability, discharge performance and power output are investigated. This report aims to play a leading role in examining the environmental stability of electrolytes in FZABs, which is critical for their practical applications.

## Introduction

The ever-growing demand for wearable and portable electronic devices as well as burgeoning energy and environmental concerns has been driving forces for advanced flexible energy-storage systems with high energy density and high economic efficiency (Su et al., 2017; Ding et al., 2019; Fan et al., 2019). To meet these requirements, the flexible zinc–air battery (FZAB), which is composed of a zinc anode, a semi-solid electrolyte and an air cathode, is a promising energy-storage technology known for its remarkably high theoretical energy density, environmental benignancy, and low cost (Xu et al., 2015a; Chen et al., 2018; Li et al., 2019). Recent progress in FZAB research has been demonstrated with polymer-based ZABs in various structures that can withstand continuous mechanical bending (Fu et al., 2016a). One of the essential components in FZABs is polymer electrolyte, which consists of a polymer matrix and the electrolyte serving as the medium to transport the ions involved in the charge–discharge cycle of the battery (Meyer, 1998). Polymer electrolytes provide the advantages of flexibility, compactness, freedom from leakage and ease of handling (Choudhury et al., 2009).

Poly(vinyl alcohol) (PVA) has been the most widely used polymer host for FZABs owing to its high chemical stability and simple fabrication process (Kim et al., 2016). PVA–KOH gel polymer electrolytes (GPEs) comprising PVA and KOH aqueous electrolyte are commonly used semi-solid electrolytes for assembling sandwich and cable-like FZABs. For example, Fu et al. fabricated a FZAB with a laminated structure by sandwiching a PVA–KOH GPE between a catalyst-loaded air electrode and a zinc-film electrode (Fu et al., 2015). The obtained battery was mechanically robust, exhibited good rechargeability and cycling stability and could be bent at different bending angles without any performance loss. Qu et al. developed a planar ZAB with electrodes, a PVA–KOH GPE, copper circuits and a rubber substrate that was assembled by a layer-by-layer method and could be stretched up to 100% (Qu et al., 2017). Furthermore, Li et al. assembled a fiber-shaped ZAB by immersing a chiffon-band-twisted zinc wire into a PVA–KOH GPE followed by complete air electrode wrapping (Li et al., 2018). The resultant FZAB could be knitted into clothes and had stable performance under various severe deformations.

To date, extensive research has focused on the development and optimization of electrode and electrocatalyst materials, as well as the battery configuration design (Song et al., 2017; Cai et al., 2018). Several reports have developed novel polymer electrolytes, such as quaternary ammonium functionalized cellulose membranes, quaternary ammonium functionalized nanocellulose/graphene oxide membranes, poly(ethylene oxide)–PVA-based polymer electrolytes, and poly(acrylic acid)-based polymer electrolytes (Xu et al., 2015b; Cheng et al., 2016; Fu et al., 2016b; Zhang et al., 2016, 2018; Guan et al., 2017). However, very few studies have focused on the influence of the environment on the electrolyte and the corresponding battery performance. Due to the unique half-open structure of FZABs, the electrolyte is in intimate contact with the ambient air, which poses great challenges for the environmental stability of electrolytes, including dimensional stability and water and ionic conductivity retention capability. Furthermore, the stability of the electrolyte under atmospheric conditions is important for battery operation, including the cycling property, discharge and power performances.

Herein, the environmental stability of PVA–KOH GPEs for FZABs is investigated for the first time to the best of our knowledge. The commonly used PVA–KOH GPE is prepared by a simple mechanical agitation approach. The phase structure, composition and morphology of the PVA–KOH electrolyte system were characterized by X-ray diffraction (XRD), Fourier transform infrared spectroscopy (FTIR) and field-emission scanning electron microscopy (FESEM), respectively. The shape and water and ionic conductivity retention capability of the GPE were studied after different exposure time under ambient conditions. The electrochemical performances of the assembled sandwich-type FZAB using the PVA–KOH GPE were also investigated by galvanostatic charge–discharge and power output measurements. This paper aims to provide further insight into the environmental stability of conventionally used PVA–KOH electrolytes for FZABs, which is essential for their practical applications.

## Materials and Methods

### Material Synthesis

Synthesis of PVA–KOH GPE: PVA powder (3 g, molecular weight of ~195,000) and 24 mL of deionized water (DI water) were mixed and magnetically stirred for 90 min at 90°C until the solution became transparent and viscous. Three grams of KOH electrolyte (dissolved in 6 mL of DI water) was then slowly added into the solution with continuous stirring for 20 min. The obtained homogeneous viscous solution was cooled to produce a 3-mm-thick polymer film, which was cooling at −10°C for 3 h.

Preparation of the catalyst-loaded air electrode: Carbon cloth (10 × 20 mm^2^, 0.32 mm in thickness) was cut to the same size followed by sequential ultrasonic cleaning in acetone, absolute ethanol and DI water for 0.5 h each. The dried carbon cloth was then loaded with a cobalt oxide (Co_3_O_4_, 30 nm, 99.5%) catalyst slurry with a mass loading of 0.3 mg cm^−2^. The catalyst ink was prepared with Co_3_O_4_ powder (9 mg) and carbon powder (Vulcan XC-72, 21 mg) in a solvent (2.4 mL DI water, 0.6 mL isopropyl alcohol and 0.3 mL Nafion® perfluorinated resin solution).

### Material Characterization

XRD spectra were obtained with a Bruker/D8 Advanced X-ray diffractometer (Cu K_α_ radiation, 10°~60°, Bruker, USA) at a scan rate of 2° min^−1^. FTIR was performed with a Nicolet iS10 (Thermo Electron. USA) from 400 cm^−1^ to 4,000 cm^−1^ at room temperature, and FESEM was performed with a Hitachi S4800 instrument (5 kV) equipped with an energy-dispersive X-ray (EDX) analysis and elemental mapping. Before analysis, all the prepared electrolytes were dried in a freeze-dryer (LyoQuest, Spain). The photographic images were produced by a three-dimensional microscope with super wide depth of field (VHX-2000c, KEYENCE, Japan). The ionic conductivity (δ) of the PVA–KOH GPE was tested by alternating current (AC) impedance measurements with a PAR 4000 (Ametek, USA) electrochemical workstation (100 kHz~0.1 Hz, amplitude of 5 mV) and was calculated according to Equation (1):

(1) $$\begin{array}{r} \delta =\frac{L}{A\times R_{b}} \end{array}$$

where *L* represents the thickness of PVA–KOH GPE between the two stainless-steel (SS, SS316, 0.1 mm in thick) plates that was measured with a laboratory Vernier caliper; *A* refers to the area of the PVA–KOH GPE between the two SS plates, and *R*_b_ represents the bulk resistance of the PVA–KOH GPE, which was calculated from the AC impedance spectrum (point of intersection on the real axis). To speed up the test process of the ionic conductivity as a function of time, the test was conducted at 25°C and 50% RH with exposure to air.

The liquid electrolyte desorption rate of the PVA–KOH GPE was measured as follows: The prepared PVA–KOH membrane (mass *W*_0_) was exposed to ambient air (25°C, 50% RH) for different time. During this process, the weight (*W*_1_) of the PVA–KOH membrane was monitored over time. The liquid electrolyte desorption rate (*W*_t_) was calculated based on Equation (2):

(2) $$\begin{array}{r} W_{t}=\frac{W_{1}-W_{0}}{W_{0}}\times 100\% \end{array}$$

### Electrochemical Measurements

The sandwich-structure FZAB was assembled by laminating the prepared PVA–KOH GPE between a clean 10 × 20 mm^2^ zinc foil with a 0.3 mm thickness and a Co_3_O_4_-loaded air cathode. The laminated integrity of the fabricated FZAB was ensured with a breathable bandage. The charge–discharge cycling performance was measured at a current density of 3 mA cm^−3^ (normalized to the battery volume) with 20 min per cycle by a LAND CT2001A multi-channel battery testing system (China). The current density is normalized to the battery volume including a zinc film (0.3 mm in thickness), a PVA–KOH electrolyte membrane (~3 mm in thickness) and a catalyst-loaded carbon cloth (~0.32 mm in thickness) with 10 × 20 mm^2^. The current density is calculated based on the Equation (3):

(3) $$\begin{array}{r} Current\;density\;=\;\frac{Test\;current}{\;Volume\;of\;FZAB} \end{array}$$

in which the test current density is 2 mA, and the volume of FZAB can be simplified as a cuboid. The polarization curves were obtained at a scan rate of 20 mA s^−1^, and electrochemical impedance spectroscopy (EIS) was performed at a potential of 1.1 V from 100 kHz to 0.01 Hz. Both tests were measured by an Ivium Stat electrochemical workstation (Ivium Technologies, Netherlands). The specific capacity and energy density were calculated according to the volume of the assembled FZAB device (mAh cm^−3^, mWh cm^−3^) and the weight of consumed zinc (mAh g^−1^, mWh g^−1^) based on Equations (4) and (5) (Fan et al., 2019):

(4) $$\begin{array}{l} Specific\;capacity\\ \;=\;\frac{\;I\;\times \;t}{\;Volume\;of\;FZAB\;or\;the\;weight\;of\;consumed\;zinc} \end{array}$$

(5) $$\begin{array}{l} Energy\;density\\ \;=\;\frac{I\;\times \;t\;\times \;U}{Volume\;of\;FZAB\;or\;the\;weight\;of\;consumed\;zinc} \end{array}$$

where *I, t*, and *U* represent the discharge current, the service hours and the average discharge voltage, respectively.

## Results and Discussion

The structure and composition of the prepared PVA were characterized by XRD and FTIR. The XRD pattern of the pure PVA is shown in Figure S1 and demonstrates a typical semi-crystalline structure. Pure PVA exhibits a high intensity peak at a 2θ value of 19.5°, corresponding to its (101) plane. Two low intensity peaks at 2θ values of 23° and 41° correspond to the (200) and (111) planes of pure PVA, respectively (Assender and Windle, 1998; Zhu et al., 2013). The FTIR spectrum of the pure PVA (Figure S2) was obtained in the range of 4,000–400 cm^−1^ at room temperature. The intense band between 3,200 and 3,500 cm^−1^ is associated with the stretching vibration of the hydroxyl groups (O–H stretching) of PVA, which are related to intramolecular and intermolecular hydrogen bonds (Qiao et al., 2010). In addition, the polymer matrix is identified by the absorption peaks at 2,942, 2,910, 1,378, 1,333, 1,093, and 853 cm^−1^, which correspond to C–H asymmetric stretching, C–H symmetric stretching, C–H bending, C–H wagging, C–O stretching, and C–H rocking, respectively (Gao and Lian, 2013).

**Figure 1:**
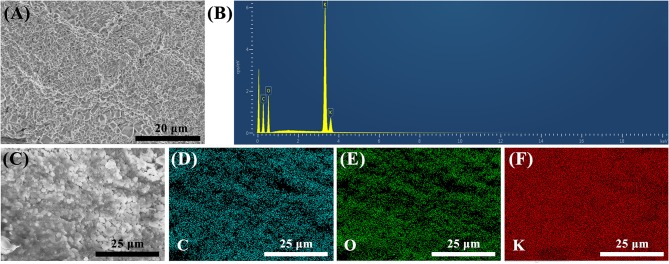
**(A)** FESEM image. **(B)** EDX analysis, **(C–F)** elemental mapping of C, O, K of PVA–KOH.

The FESEM image of the surface morphology of PVA–KOH GPE is shown in Figure 1A. The sample was freeze-dried with a laboratory freeze-dryer before characterization. The PVA–KOH GPE is a compact, homogeneous and dense material. The elemental composition of the as-prepared dried PVA–KOH GPE is identified by EDX analysis (Figure 1B), which is composed of C, O, and K elements. Furthermore, as shown in the elemental mapping images (Figures 1C–F), C, O, and K are observed clearly and dispersed evenly, indicating the effective cross-linking in the PVA–KOH GPE. Moreover, the discernable bright spots in the FESEM image are KOH particles that are precipitated from the dried GPE due to the water loss in the freeze-drying process.

**Figure 2:**
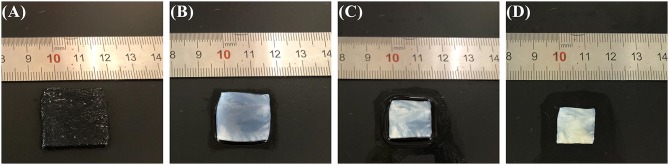
Photographs of PVA–KOH after exposure to air for **(A)** 0 h, **(B)** 6 h **(C)** 12 h, and **(D)** 24 h at 25°C and 50% RH.

ZABs are known for their extremely high theoretical energy density owing to their unique half-open structure and ability to consume oxygen from the air (Zhang et al., 2016). As the ionic transport medium between the electrodes, the electrolyte is half-exposed to ambient air, which poses a great challenge to its atmospheric dimensional stability and water and ionic conductivity retention capabilities. Among them, the dimensional stability of semi-solid electrolytes plays a critical role in the battery performance of sandwich-like FZABs and contributes to the integration of the battery to avoid displacement between the electrolytes and electrodes during charge–discharge cycles under bending conditions. Electrolyte–electrode displacement will inevitably degrade the battery cycling, discharge, rate, and polarization performances. Figure 2 shows photographs of the PVA–KOH GPE after exposure to air for 0, 6, 12, and 24 h at 25°C and 50% RH. The PVA–KOH GPE is transparent in the initial state, and its square area was measured to be 23 × 23 mm^2^ (Figure 2A). After exposure to air for 6 h, the PVA–KOH GPE tends to become opaque and shrinks to 20 × 20 mm^2^ (Figure 2B) as a result of severe water loss due to its poor water retention property. The PVA–KOH GPE continues to shrink to 15 × 15 mm^2^ after 12 h and is completely dry (13 × 13 mm^2^) after 24 h (Figures 2C,D). Furthermore, the PVA–KOH GPE membrane develops an orange color upon increasing its exposure time in ambient air owing to dehydration oxidation of PVA in air (Chandramouli et al., 1998; Fu et al., 2010).

**Figure 3:**
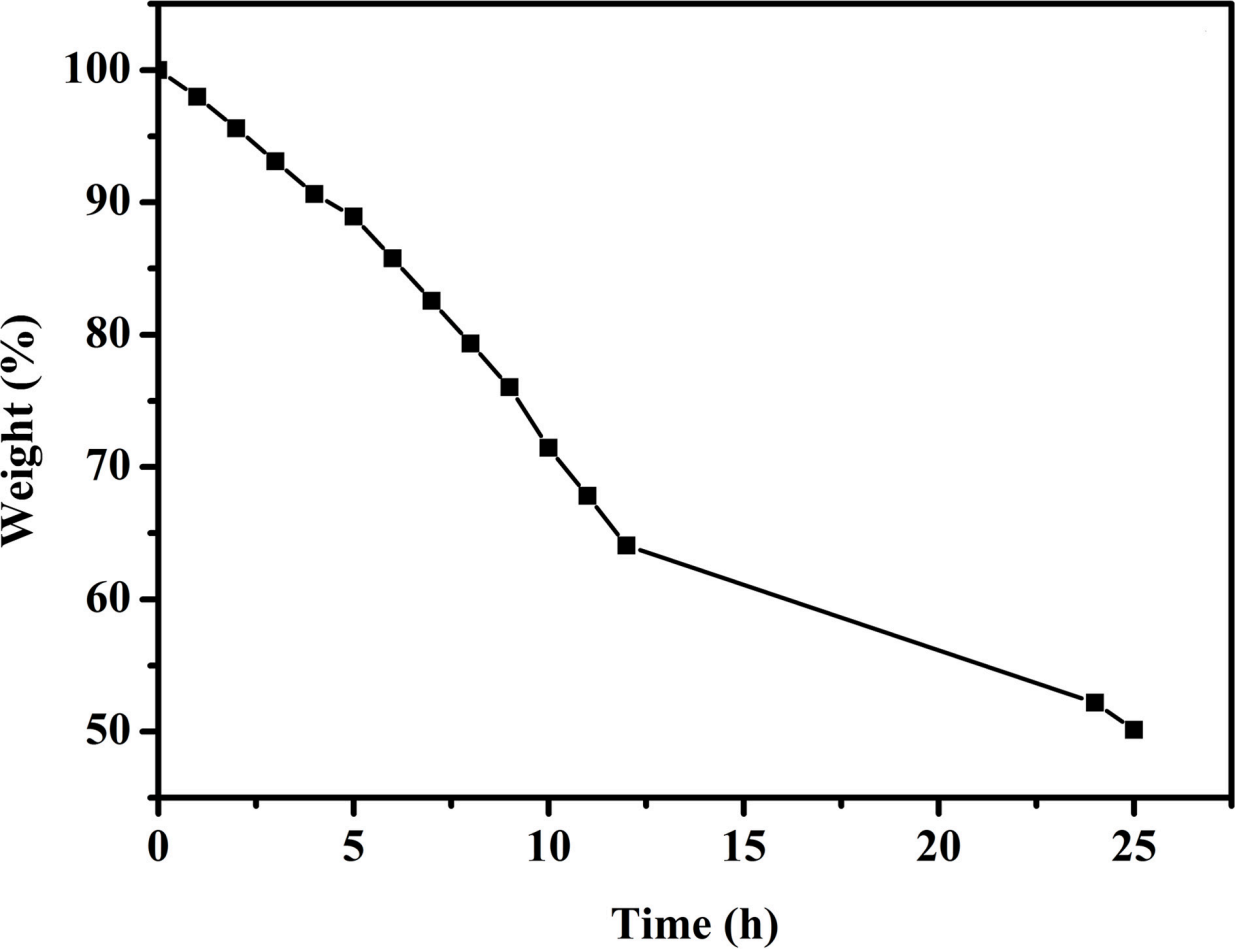
Liquid electrolyte desorption of PVA–KOH as a function of time under ambient air (25°C and 50% RH).

As the ionic conducting medium, the GPE consists of a polymer matrix and an aqueous electrolyte or a solvent with a dissolved conducting salt. The ionic conductivity of GPEs is achieved by the transport of salt ions through water or the solvent (Zhong et al., 2015). Therefore, water plays a significant role in the transport of ions for the PVA–KOH GPE, which consists of a PVA polymer matrix and KOH aqueous electrolyte. As an essential property of GPEs, the liquid electrolyte retention capability should be of great concern because high-performance FZABs benefit from the excellent electrolyte retention ability of GPEs, which maintain their high hydroxide-ion conductivity over time. Figure 3 shows the liquid electrolyte desorption of PVA–KOH as a function of time under ambient air (25°C and 50% RH) and the ratio of the weight lost to the initial weight. After exposure to air for 6 h, the PVA–KOH membrane shows a weight loss ratio of ~15% and that increases to ~35% (12 h) and ~50% (24 h).

**Figure 4:**
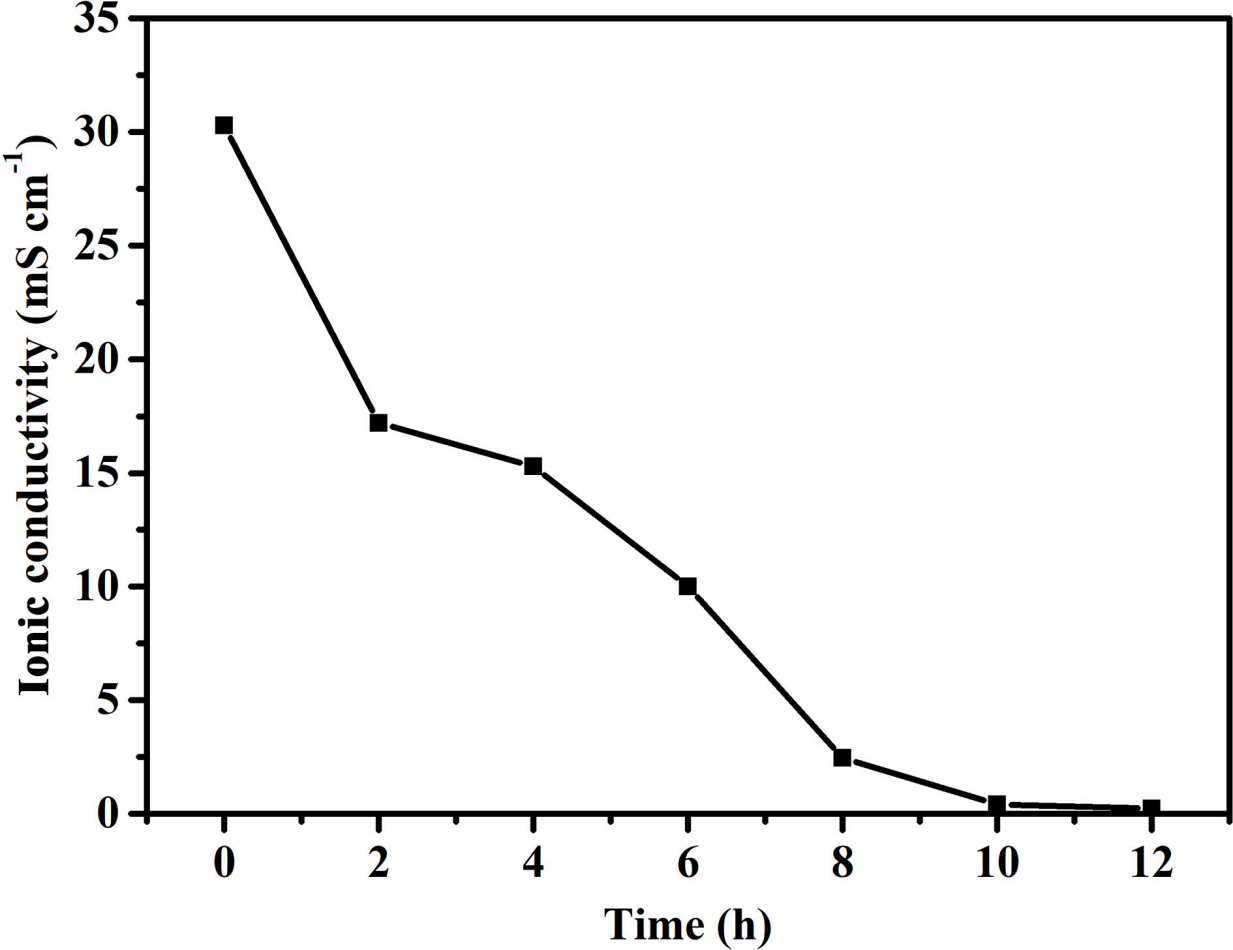
Ionic conductivity of PVA–KOH as a function of time under ambient air (25°C and 50% RH).

The ionic conductivity of electrolytes is a key parameter since the operation of FZABs relies on two important pathways, ion and electron conduction, in which ionic conductivity has a significant impact on the battery voltage, cycle life, discharge performance, and power output of FZABs. As mentioned above, the inevitable water loss of ZABs with half-open structures will have a major influence on the ionic conductivity of the PVA–KOH GPE, which primarily depends on ion transport within water. The influence of the air exposure time on the ionic conductivity of the PVA–KOH GPE was investigated, and Figure 4 shows its ionic conductivity as a function of time under ambient air (25°C and 50% RH). The PVA–KOH GPE shows the highest ionic conductivity, up to 30.3 mS cm^−1^, in its initial state. After exposure to ambient air for 6 h, the ionic conductivity of the prepared electrolyte membrane decreases to ~10 mS cm^−1^ and continues to decrease to ~1 × 10^−4^ S cm^−1^ after 12 h. In conclusion, the ion conduction of the PVA–KOH GPE exhibits a decreasing trend owing to inevitable liquid electrolyte loss under air exposure conditions, which is consistent with the liquid electrolyte desorption results (Figure 3).

**Figure 5:**
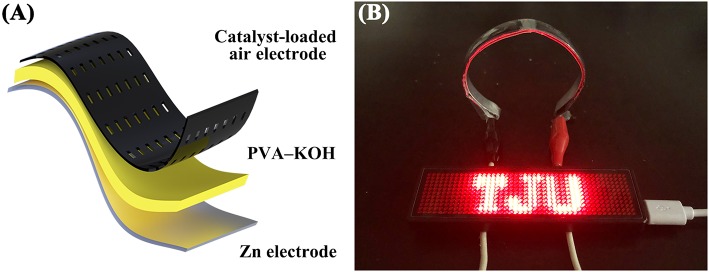
**(A)** Schematic diagram of the FZAB. **(B)** Photograph of a red LED screen powered by two FZABs in series.

**Figure 6:**
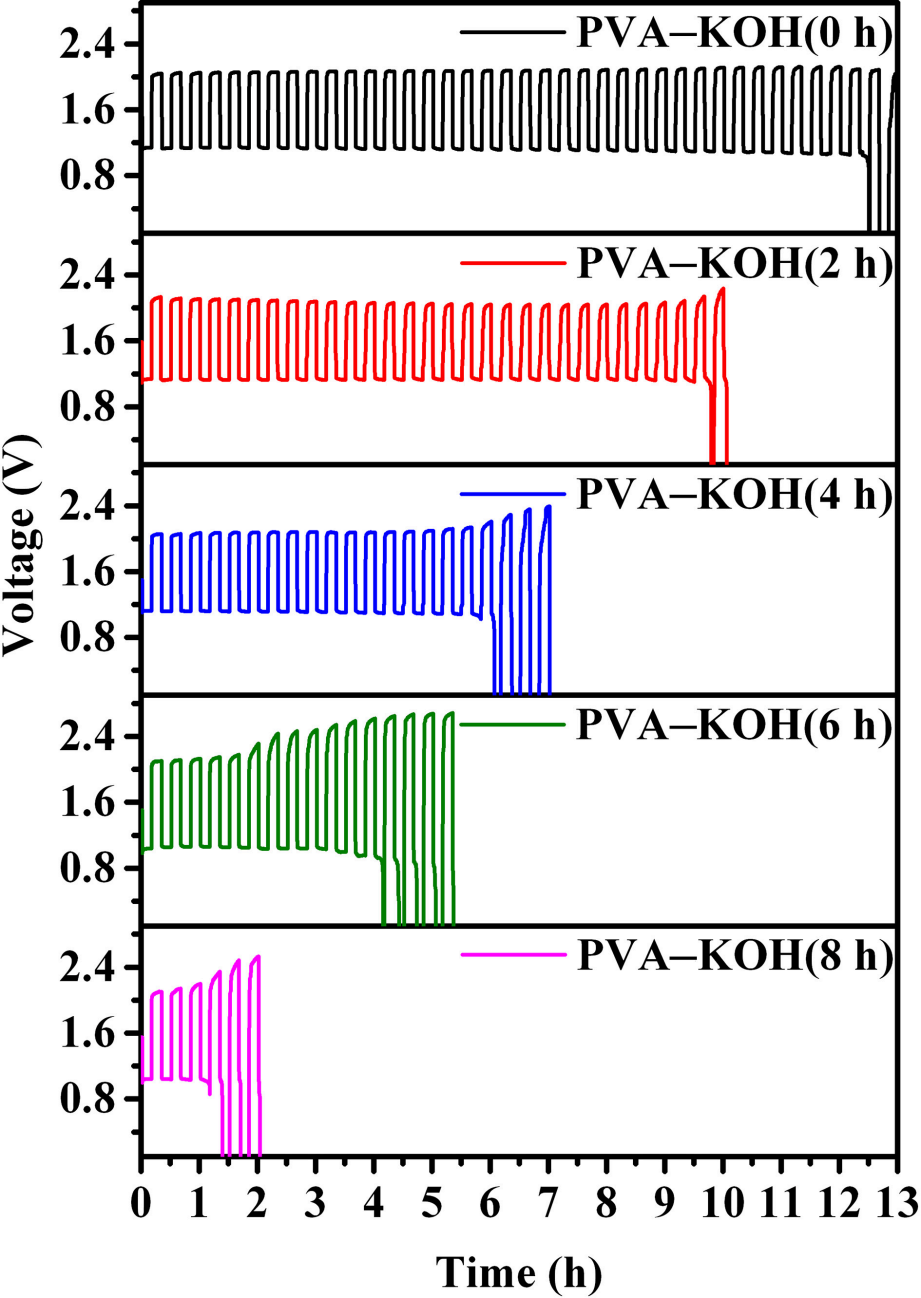
Galvanostatic charge–discharge tests of the PVA–KOH GPE-based FZAB at a current density of 3 mA cm^−3^ and 20 min per cycle.

To further investigate the practical performance of the prepared PVA–KOH GPEs in a FZAB under exposure to air, a FZAB with a sandwich structure was assembled by a layer-by-layer method. The fabricated FZAB was obtained by laminating the PVA–KOH membrane between a zinc anode and a commercial Co_3_O_4_-loaded air cathode (Figure 5A). The as-assembled FZAB shows excellent bendability and can be shaped into a semicircle while also powering a light-emitting diode (LED) screen (Figure 5B). The galvanostatic charge–discharge measurement of the PVA–KOH GPE-based FZAB at a current density of 3 mA cm^−3^ with 20 min per cycle is shown in Figure 6. The FZAB with the fresh PVA–KOH GPE shows good cycling stability and rechargeability for 12.5 h (37.5 cycles). After exposure to ambient air for 2 h, the cycling time of the assembled FZAB decreases to 9.8 h (29.5 cycles). With increasing air exposure time (4, 6, and 8 h), the cycling life of the FZAB decreases to 6, 2 and 1 h, respectively. In addition, owing to the relatively high ionic conductivity and good electrolyte–electrode interface contact (Figure S3A), the FZAB using the fresh PVA–KOH GPE exhibits a relatively small voltage gap of 0.95 V with a discharge potential of 1.15 V and a charge potential of 2.1 V (round-trip efficiency of 54.8%). The voltage gap and the round-trip efficiency of the PVA–KOH GPE-based FZAB after exposure to air for 2, 4, 6, and 8 h are 1.01 V and 52.4%, 1.0 V and 52.3%, 1.08 V and 49.3%, and 1.12 V and 47.9%, respectively. The voltage gap shows an increasing trend and the round-trip efficiency decreases with increasing exposure time, which is caused by water loss, ionic conductivity degradation and poor interfacial contact between the PVA–KOH membrane and the electrode (Figures S3B–E, S4).

**Figure 7:**
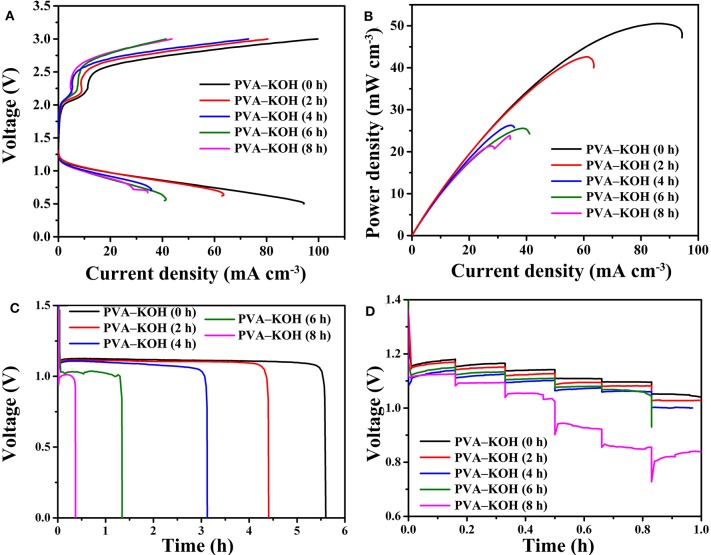
**(A)** Polarization curves, **(B)** power density curves, **(C)** galvanostatic discharge performances (3 mA cm^−3^), and **(D)** rate performances (0.75, 1.5, 3, 6, 7.5, and 15 mA cm^−3^) of the FZABs based on PVA–KOH GPEs.

The polarization performance and discharge power density curves of the fabricated PVA–KOH GPE-based FZABs after exposure to air for 0, 2, 4, 6, and 8 h are presented in Figures 7A,B. As seen from the figures, the FZAB with the fresh PVA–KOH GPE shows a maximum charge current density of 99.1 mA cm^−3^, a maximum discharge current density of 94 mA cm^−3^ and a peak power density of 50.5 mW cm^−3^. After exposure to air for 2 h, the assembled FZAB shows a maximum charge current density of 80.4 mA cm^−3^, a maximum discharge current density of 63.4 mA cm^−3^ and a peak power density of 42.6 mW cm^−3^. All of these values exhibit a decreasing trend; after 8 h of exposure, the maximum discharge current density and the peak power density decrease to 34.3 mA cm^−3^ and 23.8 mW cm^−3^, respectively. The galvanostatic discharge performance of the FZABs using the PVA–KOH GPE after exposure to air for 0, 2, 4, 6, and 8 h was tested at 3 mA cm^−3^ under ambient air (Figure 7C). The discharge plateau of the FZAB in the fresh state is 1.15 V, which is higher than that obtained using PVA–KOH GPE after 2, 4, 6, and 8 h of exposure. In addition, the discharge time of the fresh PVA–KOH GPE-based FZAB is 5.6 h, and as the air exposure time increases to 2, 4, 6, and 8 h, the discharge time decreases to 4.4, 3.1, 1.4, and 0.4 h, respectively. The specific capacity of the FZABs fabricated using the PVA–KOH GPE, which is calculated based on the discharge performance, is normalized to the battery volume and the amount of zinc anode consumed. As measured, the FZAB using the fresh PVA–KOH GPE could deliver the highest specific capacity of 15.5 mAh cm^−3^ (466.7 mAh ${\text{g}}_{\text{Zn}}^{-1}$) and energy density of 17.2 mWh cm^−3^ (517.4 mWh ${\text{g}}_{\text{Zn}}^{-1}$) at 3 mA cm^−3^, and both of these values decrease with an increase in exposure time. The rate performance of the PVA–KOH GPE-based FZAB at different current densities (0.75, 1.5, 3, 6, 7.5, and 15 mA cm^−3^) is presented in Figure 7D. The discharge plateau of the FZAB with the fresh PVA–KOH GPE decreases from 1.18 V (0.75 mA cm^−3^) to 1.04 V (15 mA cm^−3^), and the decrease of 0.14 V is superior to that observed with the PVA–KOH GPE exposed to air for 2, 4, 6, and 8 h. The freshly prepared PVA–KOH GPE shows a relatively high ionic conductivity and good interfacial property and can be used to fabricate an FZAB that exhibits better cycle stability, discharge property, rate, polarization performance, and power output than FZABs fabricated with GPEs exposed to air for several hours. This result indicates that the commonly used PVA–KOH GPE can be applied as the ionic conductor for FZABs within several hours of preparation. However, the unique half-open structure of FZABs suffers from severe water loss, leading to the degradation of ion transport and severe shape changes in the PVA–KOH GPE with increasing exposure time. Therefore, a sufficient ionic conductivity with a high water and electrolyte retention capability as well as a good electrolyte–electrode interfacial contact are important for mitigating ohmic polarization and promoting the resulting reaction kinetics of FZABs.

## Conclusion

In summary, the commonly used PVA–KOH GPE was investigated in terms of its environmental stability, including its dimensional stability and water and ionic conductivity retention capability in ambient air. With the utilization of this PVA–KOH GPE, an FZAB was fabricated with a sandwich-like structure, and the cycling stability, discharge performance and power output of the FZAB were deeply studied. The fresh PVA–KOH GPE is a transparent and soft polymer material with a relatively high ionic conductivity (30.3 mS cm^−1^), and the assembled FZAB showed a cycling life of 12.5 h, a discharge time of 5.6 h and a relatively high power output. With the increase in the exposure time in ambient air, the PVA–KOH GPE suffered from an obvious shape change and severe loss of water and ionic conductivity, which resulted in degradation of the cycling property, discharge performance and power output of the fabricated FZABs. To the best of our knowledge, this is the first report to investigate the environmental stability of PVA–KOH GPEs for FZABs, which is important for their practical application.

## Data Availability Statement

The datasets generated for this study are available on request to the corresponding author.

## Author Contributions

XF, WH, and CZ contributed conception and design of the study. XF organized the database and wrote sections of the manuscript. JL, JD, YD, and XH performed the statistical analysis. XF wrote the first draft of the manuscript. All authors contributed to manuscript revision, read, and approved the submitted version.

### Conflict of Interest

The authors declare that the research was conducted in the absence of any commercial or financial relationships that could be construed as a potential conflict of interest.
