# Supplementary material for: Investigation of the Environmental Stability of Poly(vinyl alcohol)–KOH Polymer Electrolytes for Flexible Zinc–Air Batteries
Source: Front Chem. 2019 Oct 22;7:678. doi: 10.3389/fchem.2019.00678 (PMC6817501; doi:10.3389/fchem.2019.00678)
Supplement: Supplementary file 1 [file Table_1.DOCX]

**Supporting Information**

**Investigation of the Environmental Stability of Poly(vinyl alcohol)–KOH Polymer Electrolytes for Flexible Zinc–Air Batteries**

**Xiayue Fan^1^, Jie Liu^1^, Jia Ding^1^, Yida Deng^2^, Xiaopeng Han^2^, Wenbin Hu^1,2,3^, Cheng Zhong^1,2,3^**^*^

^1^Key Laboratory of Advanced Ceramics and Machining Technology (Ministry of Education), School of Materials Science and Engineering, Tianjin University, Tianjin 300072, China.

^2^Tianjin Key Laboratory of Composite and Functional Materials, School of Materials Science and Engineering, Tianjin University, Tianjin 300072, China.

^3^Joint School of National University of Singapore and Tianjin University, International Campus of Tianjin University, Binhai New City, Fuzhou 350207, China

*** Correspondence:**

Cheng Zhong

cheng.zhong@tju.edu.cn

**Keywords: poly(vinyl alcohol), KOH, gel polymer electrolyte, environmental stability, flexible zinc–air batteries.**





**Figure S1.** XRD pattern of the prepared PVA.


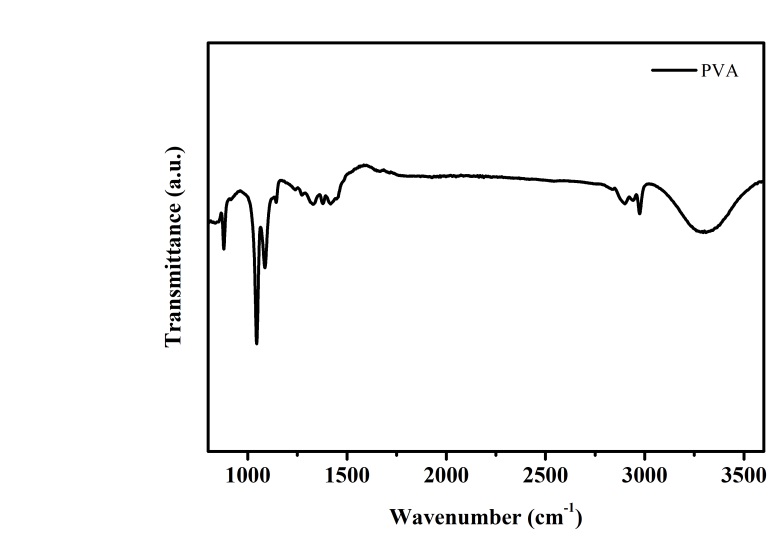


**Figure S2.** FTIR spectrum of the prepared PVA.

**
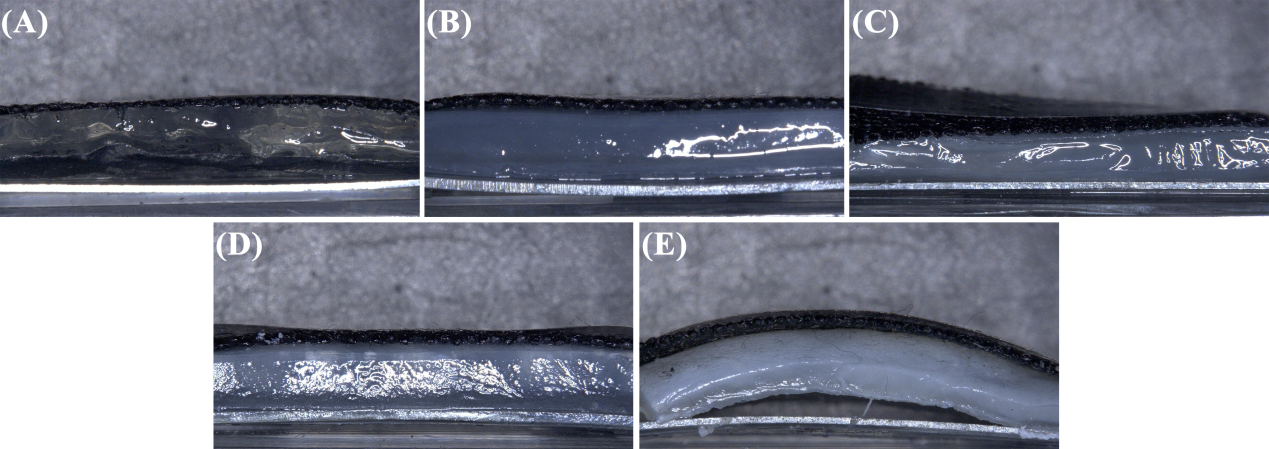
**

**Figure S3.** Cross-section photographic images of FZABs using PVA–KOH GPE after exposure to air for **(A)** 0 h, **(B)** 2 h **(C)** 4 h, **(D)** 6 h, and **(E)** 8 h at 25 °C and 50% RH.

**
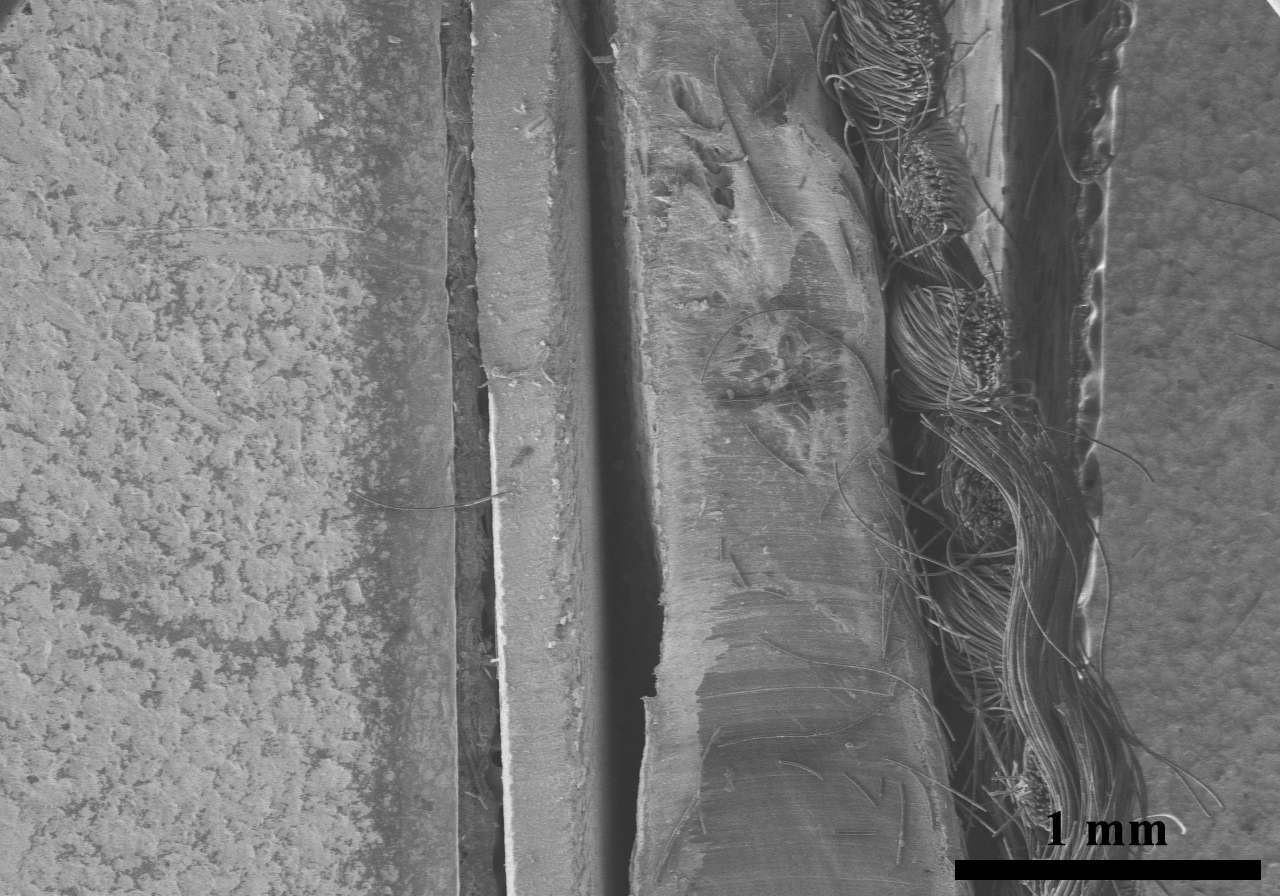
**

**Figure S4.** Cross-section FESEM of FZAB.
